# Magnesium, zinc, aluminium and gallium hydride complexes of the transition metals†
†Electronic supplementary information (ESI) available. See DOI: 10.1039/c6cc05702k


**DOI:** 10.1039/c6cc05702k

**Published:** 2017-01-03

**Authors:** Michael J. Butler, Mark R. Crimmin

**Affiliations:** a Department of Chemistry , Imperial College London , South Kensington , London SW7 2AZ , UK . Email: m.crimmin@imperial.ac.uk ; Tel: +44 (0)2075942846

## Abstract

The preparation and applications of heterobimetallic complexes continue to occupy researchers in the fields of organometallic, main group, and coordination chemistry. This interest stems from the promise these complexes hold as precursors to materials, reagents in synthesis and as new catalysis. Here we survey and organise the state-of-the-art understanding of the TM–H–M linkage (M = Mg, Zn, Al, Ga). We discuss the structure and bonding in these complexes, their known reactivity, and their largely unrealised potential in catalysis.

## Abstract 2

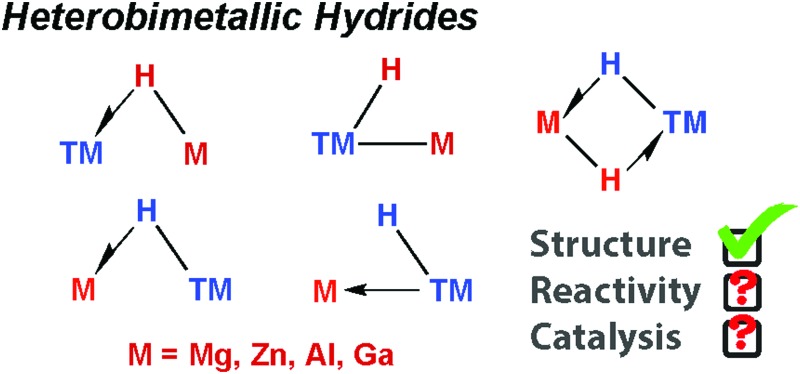
Here we survey and organise the state-of-the-art understanding of the TM–H–M linkage (M = Mg, Zn, Al, Ga). We discuss the structure and bonding in these complexes, their known reactivity, and their largely unrealised potential in catalysis.

## 1. Introduction

**Fig. 1:**
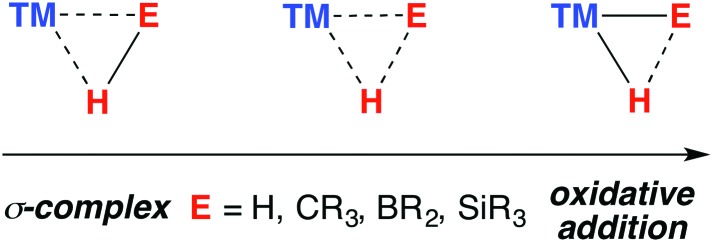
The continuum between σ-complex and oxidative addition for the coordination of E–H bonds to a transition metal.

The catalytic practices of C–H bond functionalisation, dehydrocoupling (for hydrogen storage), hydroboration and hydrosilylation are all attractive prospects for the future chemical economy. The modern development in these methodologies continues to be enhanced by the perception of borane and silane σ-complexes as intermediates in reaction mechanisms (Fig. 1).

A σ-complex can be described as an η^2^-binding of the σ-E–H bond to a transition metal centre (E = C, Si, B, H).^1^,^2^ Along with dihydrogen complexes,^3^–^6^ σ-silanes are the most comprehensively studied type of this bonding mode.^7^–^12^ While the latter appear as potential intermediates in alkene hydrosilylation *via* the Chalk–Harrod mechanism,^13^,^14^ the former bear significance for a range of industrially relevant hydrogenation reactions.^15^,^16^ In C–H borylation, stabilisation of catalytic intermediates by a TM–H–B (TM = transition metal) interaction has been supported by significant experimental mechanistic studies.^17^–^19^

A 3-centre 2-electron interaction, the η^2^-ligation of E–H to TM can be viewed within the Dewar–Chatt–Duncanson model and interpreted as a combination of: (i) donation of the σ-electrons in the E–H bond to a vacant orbital on the transition metal and (ii) back-donation from the metal into the σ*-orbital of the same E–H bond. The resulting σ-E–H adduct represents an intermediate along the oxidative addition reaction coordinate (Fig. 1) – part of a continuum of bonding descriptions between free E–H and E–TM–H. It is clear that the nature of the bonding in TM–H–E containing complexes is a tuneable property, being a consideration of the symmetry and energy of the frontier orbitals of the transition metal fragment along with the substituents on, and nature of, E.

A fundamental question that arises when considering this model, is: what happens when C, B and Si are replaced by metallic main group elements such as Mg, Zn, Al, or Ga? The increased ionic contribution within the TM–H–M interaction will necessarily give another dimension to the bonding description. As with silicon, the ability of these elements to expand their coordination sphere leads to the possibility of forming additional bonding interactions with existing ligands on the TM fragment. Furthermore, for the heavier group 13 elements the formation of low-valent M^I^ ligands through manifestation of the inert-pair effect becomes an important consideration.

**Fig. 2:**
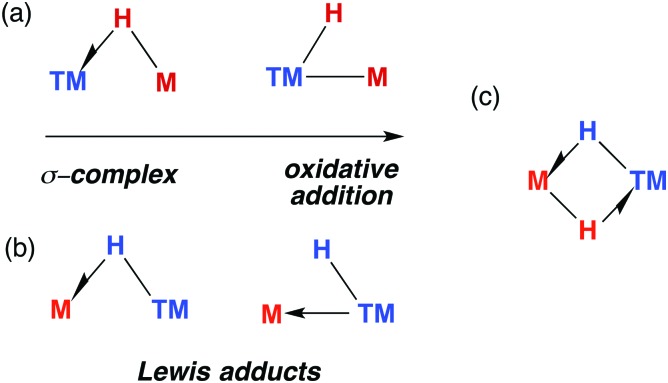
(a) The continuum for addition of M–H to a transition metal. (b) Lewis adducts between a TM hydride and M, (c) combination of interactions.

The TM–H–M motif is one way of adjoining two metal centres bearing at least one reactive hydride ligand (Fig. 2a). This motif can also be obtained by coordinating a transition metal hydride to a neutral main group metal fragment (Fig. 2b), and multiply bridged species formed by a combination of the two aforementioned donor–acceptor interactions (Fig. 2c).

Herein we survey the known heterobimetallic complexes of transition metal and main group hydrides (M = Mg, Zn, Al, Ga). The coordination chemistry of heterobimetallic transition metal hydrides,^20^–^22^ and of Al, Ga, In and Zn-based ligands at transition metal centre have been summarised previously.^23^–^26^ To focus the discourse, a loose definition ‘heterobimetallic hydride complexes’ is employed. The complexes mostly fit these criteria: (a) crystallographically characterised; (b) 1 : 1 ratio of transition : main group metal; (c) the absence of TM···TM interactions; (d) a hydride ligand in a bridging role. The survey is arranged: TM←H–M, TM–H→M, TM–H_*n*_–M (*n* > 1).

The half-arrow notation and covalent bond classification advocated by Green, Green and Parkin are used to represent the TM–H–M 3-centre 2-electron interactions.^11^ This formalism is employed as an organisational principle not as an absolute interpretation of the bonding within TM–H–M units. The line drawings are constructed from the perspective of the transition metal coordination environment in order to represent charge neutral species rather than an accurate representation of the bonding within the TM–H–M group. The literature survey is followed by discussion of the “continuum” of bonding descriptions, reactivity and the potential these complexes hold for catalysis.

## 2. σ-Complexes (TM←H–M)

**Fig. 3:**
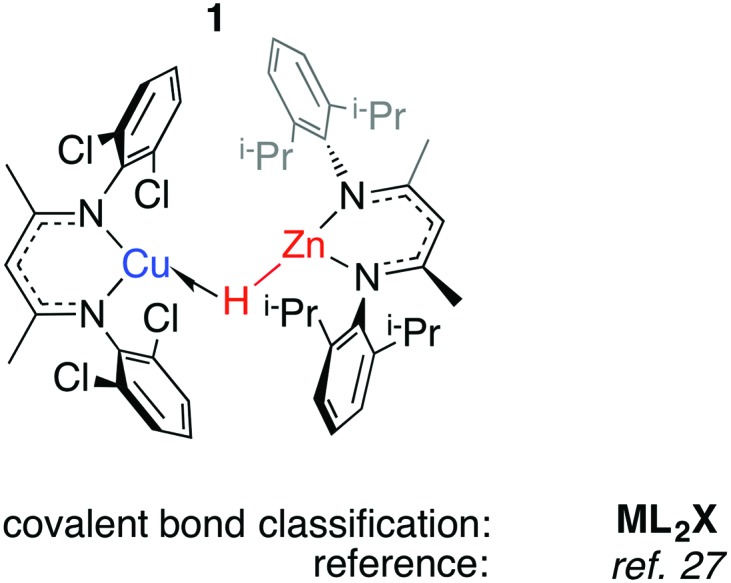
A σ-zincane complex of Cu^I^.

We have reported **1**, a Zn congener of structurally related σ-alane complexes (Fig. 3).^27^ The binding of the zinc hydride to the Cu^I^ centre is weak and reversible. In toluene or benzene solution, an equilibrium exists between the heterobimetallic complex and the η^2^-arene complex of Cu^I^. The electronic structure of the three-centre linkage has been investigated by DFT calculations. The analysis suggests that the formal L donation of the M–H σ-bond to the 4s orbital on Cu^I^ is accompanied by weak Cu→M back-donation into the M–H σ*-orbital (see Discussion section). In combination, the data allow **1** to be described as a weakly bound σ-complex.

**Fig. 4:**
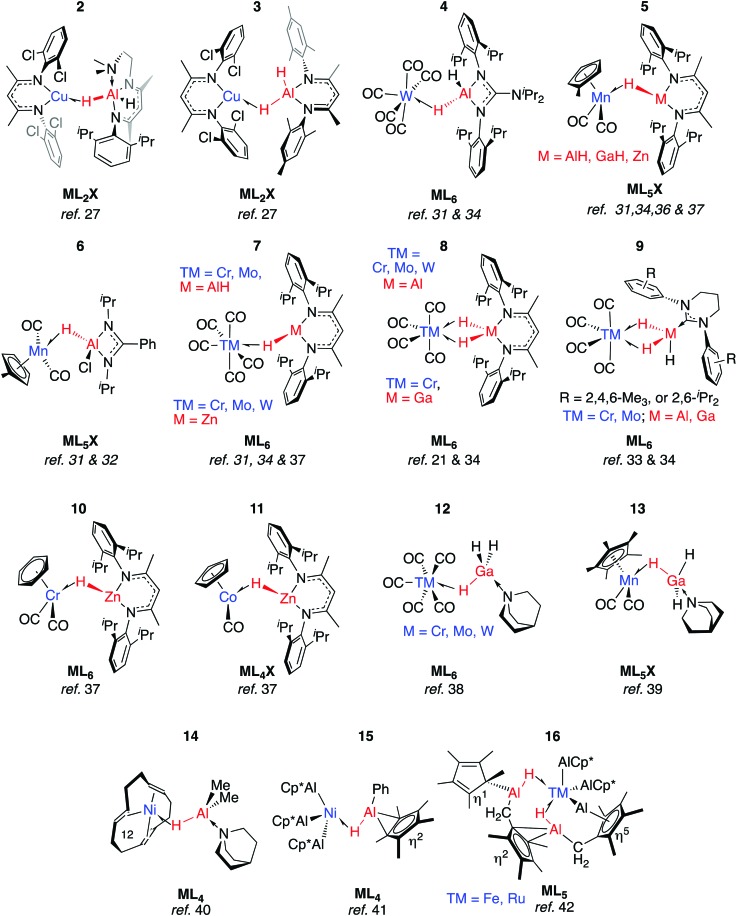
σ-Alane, σ-gallane and σ-zincane complexes of late TM.

Coordination of Al–H bonds to Cu^I^ has also been examined; complexes possessing four-coordinate and five-coordinate aluminium centres have been isolated (Fig. 4, **2** and **3**). The binding of Cu–H–Al is again weak and reversible based on solution NMR studies and crossover experiments. In DFT calculations, the coordination of exogenous ligands to the Cu^I^ fragment was found be increasingly exergonic across the series C_6_F_6_ < H–B < H–Si < C_6_H_6_ < H–Zn < H–Al – a trend that is manifest in the experimental data.^27^

In **2** the Cu···Al vector lies outside of the H–Al–H wedge – the only example of this structural feature we are aware of in main group metal σ-complexes.^27^ The ‘lee side’ coordination of the Al–H bond and very long intermetallic distance in **2** are notable. For comparison, σ-complexes of HBpin (pinacolborane) or HBcat (catecholborane) show different coordination geometries to those of four-coordinate boranes BH_3_·EMe_3_ (E = N, P).^28^,^29^ The discrepancy has been rationalised by disruption of the TM···B back-donation due to the absence of a vacant orbital of suitable energy in BH_3_·EMe_3_, be it the boron p-orbital or the σ*-orbital of the B–H bond.^28^ While steric factors are undoubtedly important, a similar effect may explain the solid state structures of **2** and **3**. DFT calculations are consistent with reduced back-donation from d^10^ Cu^I^ into the Al–H σ*-orbital of the five-coordinate species when compared to the four-coordinate analogue.^27^ Higher nuclearity species containing Cu–H–Al interactions are known and the intermetallic cluster [(Cp*AlCu)_6_H_4_] has been obtained from reaction of [Cp*Al]_4_ with [Ph_3_PCuH]_6_.^30^

Aldridge and co-workers pioneered this area of research and have isolated a series of σ-alane and σ-gallane complexes of groups 6 and 7 transition metal carbonyls.^31^–^35^ Like **2** and **3**, the structures of **4–7** contain σ-Al–H adducts and derive from ligand exchange reactions, in this case from the parent metal carbonyl under either thermal or photochemical conditions. Four-electron, η^2^:η^2^-coordination of H–Al–H (**8**–**9**) requires a 14-electron transition metal fragment, {M(CO)_4_} and necessitates displacement of two ligands from ML_6_ starting materials.^31^,^33^,^36^ Complexes **7-Cr/Al** and **8-Cr/Al** have not been separated and, like their heavier congeners, **7-Mo/Al** and **8-Mo/Al** were isolated in an approximate 9 : 1 mixture with the minor component containing the η^2^:η^2^-coordination mode.^34^ The adduct **8-W/Al** could not be obtained by photoejection of CO from [W(CO)_6_] and was finally obtained under thermal conditions using [W(CO)_4_(1,5-COD)] as a precursor (COD = cyclooctadiene).^34^ The M···Al distance in **8-W/Al** is shorter than would be expected based on the lighter members of the series, presumably due to a tighter binding of the σ-alane to the more expanded 5d orbitals of W. In contrast to the W analogue, **8-Cr/Al** may be formed directly upon heating **7-Cr/Al**: Eyring analysis and the first order kinetics of this reaction have led Aldridge and co-workers to suggest it proceeds by an associative pathway.^34^

All TM–H–Al heterobimetallic complexes characterised by Aldridge and co-workers show slow exchange between the bridging and terminal hydride ligands on the NMR timescale at ambient temperature.^31^–^34^,^36^ From structural and spectroscopic evaluation of these complexes (**4–9**), it appears that back-donation into the M–H σ* orbital is negligible. While the close TM···M contacts (especially in **9-Ga**) are short enough to hint at TM–M interaction, the four-membered ring imparted by the η^2^:η^2^-coordination mode in **8**–**9** demands such a short contact.^33^ The weaker nature of the Ga–H bonds (*cf.* Al–H) means **8-Ga** is only a minor product of the reaction of the corresponding gallane with [M(CO)_4_(1,5-COD)] as this species is unstable with respect to dihydrogen elimination (*vide infra*).^36^

Although the coordination chemistry of Zn–H–TM and Mg–H–TM groups remains underdeveloped when compared to the aluminium analogues, we have recently reported a series of σ-zincane complexes of closely related transition metal carbonyl fragments. Ligand exchange reactions readily occur under photochemical conditions and **5-Zn**, **7-Zn**, **10–11** have been isolated and crystallographically characterised.^37^

Ueno and co-workers provided evidence that the η^2^:η^2^-coordination mode is not necessary for the stabilisation of σ-gallanes, and reported **12** and **13**.^38^,^39^ These complexes were formed from displacement of THF or CO from group 6 transition metal carbonyls by GaH_3_·quinuclidine. While there is little account for TM···Ga interactions in **12**, **12-W** again contains a shorter TM···Ga separation than what would be reasoned from inspection of the solid state structures **12-Cr** and **12-Mo**.^38^,^39^ Complex **13** is noticeably similar to **6** in terms of the TM–H–M parameters. In both cases, the Cp_centroid_–Mn–H–M torsion angle is near 90° allowing for overlap of the M–H σ*-orbital with the HOMO of the Mn fragment. There are also similarities to the first reported example of a σ-alane complex. Formally a Ni^0^ species possessing a triene ligand and a coordinated alane, **14** contains an unsupported Ni–Al–H linkage that survives the substitution of the tridentate ligand with three equivalents of CO to form [(CO)_3_Ni(μ-H)AlMe_2_·quinuclidine].^40^

An alternative approach to TM–H–Al groups has been discovered by Fischer and co-workers. Addition of [Cp*Al]_4_ to transition metals with labile ligands is proposed to generate intermediates of the form [TM(AlCp*)_*n*_] which react further, effecting the inter- or intramolecular C–H activation of arenes or alkanes.^41^,^42^ Complexes **15–16** are formed through this route and possess geometries that are consistent with the σ-alanes described above (Fig. 4). For example, the Al–H distance in **15** of 1.76(3) Å lies within the range established σ-alanes. While **16** possesses elongated Al–H distances ranging from 1.88(8) to 1.89(7) Å, these are still substantially shorter than the >2.0 Å separation required to suggest oxidative addition (*vide infra*). The latter may be described as a complex containing stretched σ-alane ligands with an Al–H–TM geometry somewhere between coordination and oxidative addition.

## 3. Oxidative addition/hydride transfer (H–TM–M)

**Fig. 5:**
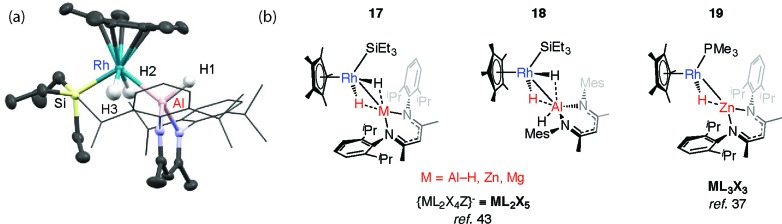
(a) The crystal structure of **17-Al**. Selected bond lengths (Å): Rh(1)–Si(1) 2.3668(7), Rh(1)–Al(1) 2.4579(7), Al(1)–H(1) 1.51(2), Al(1)–H(2) 2.13(3), Al(1)–H(3) 2.22(3), Rh–H(2), 1.47(3), Rh–H(3) 1.53(3). (b) Oxidative addition (hydride transfer) of M–H bonds to a Rh complex.

We have reported the thermal reaction of M–H bonds (M = Al, Zn, Mg) with [Cp*Rh(H)_2_(SiEt_3_)_2_] to form **17** and **18**,^43^ a reaction that is believed to proceed through the 16-electron intermediate {Cp*RhH(SiEt_3_)}. These species are different to the σ-complexes described above and data are consistent with the product of oxidative addition (Fig. 5).

In all cases the TM–M distances are within the sum of covalent radii and the M···H distances stretch to well beyond 2.0 Å. Moreover the four-legged piano-stool geometry around the Rh centre, including the *trans*-relation of the hydride ligands, is conserved when compared with silane and borane analogues, [Cp*Rh(H)_2_(SiEt_3_)(X)] (X = SiEt_3_, Bpin). The description of **18** as an oxidative addition product is supported by the significant ^1^*J*_Rh–H_ value of 40.2 Hz and terminal *ν*(Rh–H) frequency of 1966 cm^–1^. The calculated charges (NBO analysis) on Rh are significant for only the Zn and Mg analogues. As such, while these Zn and Mg complexes could be described as oxidative addition products, hydride transfer to form an is also a fair description.^43^

The structure of **17-Al**, an analogue of **18** that incorporates more sterically demanding substituents on the β-diketiminate ligand, shows a geometry with familiar *trans*-disposed hydrides, short TM–Al distance and long Al···H distances (Fig. 5). Reaction of **17-Zn** with an excess of PMe_3_ under photochemical conditions leads to the elimination of an equivalent of silane and formation of **19**. This latter heterobimetallic complex again contains a short TM–M distance and a M···H separation of greater than >2.1 Å.^37^

**Fig. 6:**
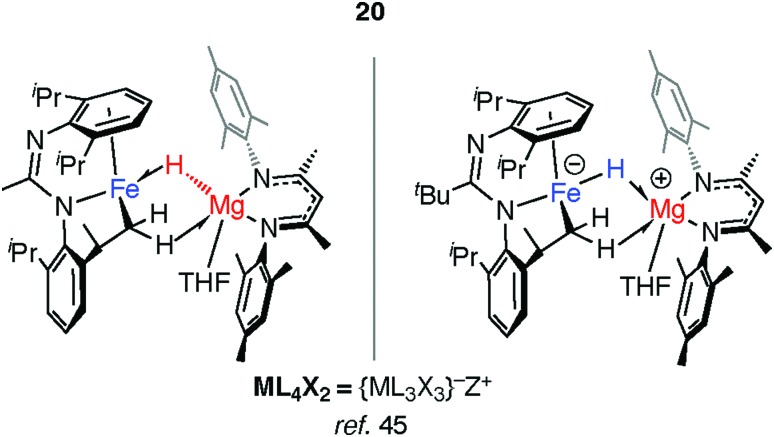
Hydride transfer to form a tightly bound ion-pair in **20**.

Mindiola and co-workers have isolated a related Fe–H–Mg complex, albeit as a minor component of a mixture formed upon reaction of EtMgCl with an iron chloride precursor.^44^ Complex **20** also results from C–H activation of the ligand (Fig. 6).^45^ While it could be assigned as an σ-Mg–H complex of Fe the long Mg···H distance and short Fe–H separation make this debatable, it is arguably closer to an ate-complex formed by a tightly bound Fe–H^–^→Mg^+^ ion-pair. Although both **20** and **17–19** can be described as the products of hydride transfer the key difference is that the latter are formed from addition of the M–H bond to the TM centre and contain a defined and quantifiable TM–M bond.

## 4. Low-valent main group ligands from dehydrogenation (TM←M)

The products of oxidative addition of M–H bonds to transition metals may only be intermediates on a path to coordinated low-valent fragments (M = Al, Ga). As the main group is descended, not only does the reduced M–H bond strength result in easier M–H “bond activation”, but the manifestation of the inert pair effect means the lower common oxidation state becomes increasingly stable.

**Fig. 7:**
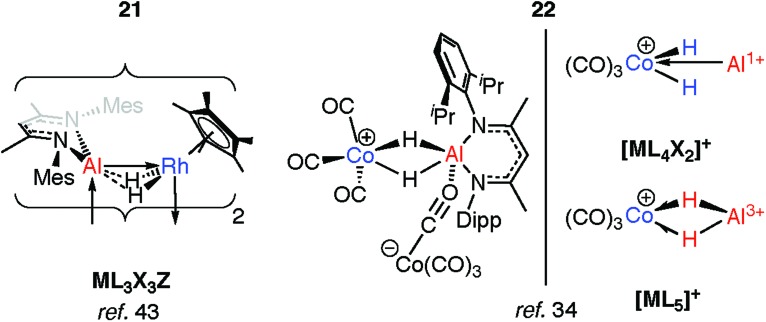
Generation of Al^I^ ligands from addition of aluminium dihydrides to TM.

Two examples of the generation of Al^I^ ligands by the dehydrogenation of aluminium dihydride precursors in the coordination sphere of a transition metal have been reported. The products of these reactions retain an undeniable H···Al interaction. The photochemical elimination of HSiEt_3_ from **19** leads to the dimer **21** (Fig. 7).^43^ Complex **21** contains a Rh_2_Al_2_H_4_ core. Supporting the argument for Al^I^ is the deviation of the alumocycle from planarity, suggestive of decreased π-donation by the N atoms into the 3p orbital of Al. This allows for Al^I^ to act as both a Z- and L-type ligand with respect to Rh.^43^ Structurally related intermetallic compounds containing Pt_2_Ga_2_H_4_,^46^ Ru_2_GaH_2_,^47^ Ru_2_Ga_2_H_4_,^48^ Ru_2_Al_2_H_4_,^48^ Co_2_H_2_Al_2_,^49^ Rh_2_Zn_2_H_2_ groups are all known,^50^,^51^ as are higher nuclearity species in which multiple main group fragments act as ligands for the transition metal (see ESI,† Fig. S1).^52^–^57^ The work on cluster complexes supported by organozinc, organoaluminum and organogallium ligands has been reviewed before.^23^–^26^

Coordination of a related aluminium dihydride to a 14-electron {Co^I^(CO)_3_}^+^ synthon gives **22** (Fig. 7).^34^ This latter species appears to be a product of simultaneous addition of both Al–H bonds to Co. The bridging Al···H distances of 1.92(3)–1.98(3) Å in **22** are not as long as those found in the oxidative addition products **17-Al** or **18** of 2.0–2.2 Å but are significantly longer than those found in σ-complexes of the same aluminium species which typically range from 1.6–1.8 Å. As with **21**, the aluminium centre receives additional electron density, here by end-on coordination of an isocarbonyl ligand of the formally anionic {Co(CO)_4_} moiety. The electronic structure of **22** lies somewhere between the bis σ-complex and the dehydrogenated Co^III^/Al^I^ species (Fig. 7).

**Fig. 8:**
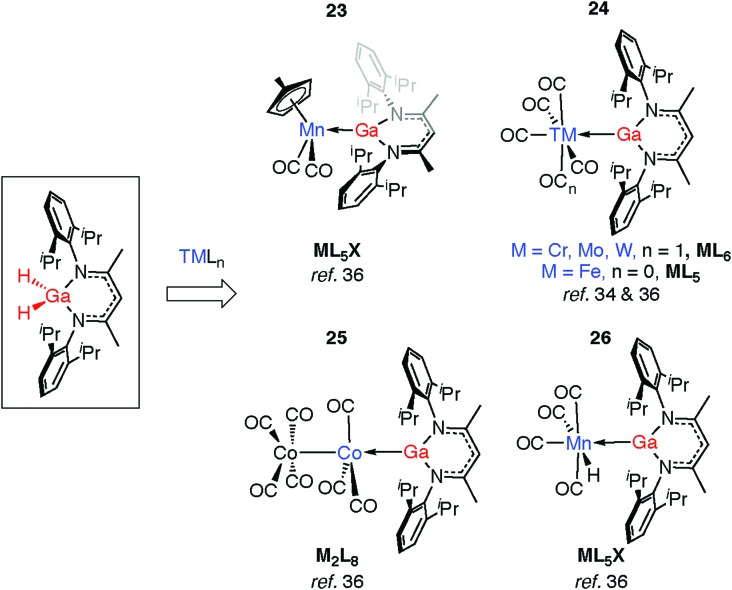
Generation of Ga^I^ ligands from addition of gallium dihydrides to TM.

Complexation of gallanes to late transition metal carbonyls (TM = Cr, Mo, W, Mn, Fe, Co) has been shown to lead to the formation of Ga^I^ ligands through spontaneous or photoinduced extrusion of dihydrogen (Fig. 8).^34^,^36^ Complexes **23** and **24** are formed from addition of gallanes to [(η^5^-C_5_H_4_Me)Mn(CO)_3_] and [TM(CO)_*n*_] (TM = Cr, Mo, W, *n* = 6; TM = Fe, *n* = 5) precursors. A bimetallic reaction intermediate is proposed, from which 1,2-elimination of dihydrogen occurs. Reactions employing 34-electron carbonyls [Mn_2_(CO)_10_] or [Co_2_(CO)_8_] in place of the 18-electron [M(CO)_*n*_] complexes proceed similarly. For Co, oxidative addition is followed by H_2_ elimination across the TM–M bond to give **25**. For Mn, oxidative addition of the Ga–H bond is followed by reductive elimination of H–TM(CO)_4_ and α-migration of the remaining hydride from Ga to Mn to form **26** (see Discussion, Fig. 23).^36^ The proposed intermediate of the latter reaction contains a Mn–Ga–H group and finds experimental support from the work of the groups of both Driess and Fischer who have reported complexes containing Fe←Ga–H,^58^ and TM←M–H moieties (TM = Cr, Mo, Zn; M = Al, Ga).^59^–^61^

## 5. Hydride-bridged Lewis adducts (TM–H→M)

### Late transition metal adducts

Lewis acidic main group metal centres without hydride substituents may be coordinated by transition metal hydrides in two different ways: (i) through a direct metal–metal interaction from donation of d-electrons of the TM to the main group metal (Z-type ligand) or (ii) through 3-centre 2-electron hydride bridges of the form TM–H→M (Fig. 2). Elimination reactions may result from these adducts provided the p*K*_a_ of the hydride is low enough and the metal alkyl (or aryl) basic enough to effect alkane (or arene) elimination.

**Fig. 9:**
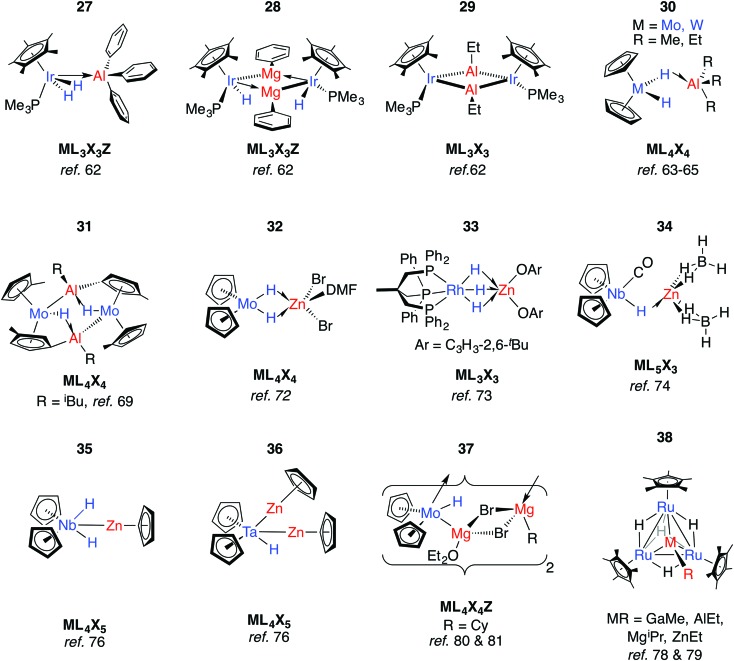
Late transition metal adducts.

For example, Andersen and Bergman have reported the reaction of [Cp*Ir(H)_2_(PMe_3_)] with a series of organo-aluminium and -magnesium compounds (Fig. 9).^62^ Coordination of Ph_3_Al to form **27** results in the widening of the H–Ir–H angle by 20° and signifies dominant TM–M bonding. In contrast, the reaction of [Cp*Ir(H)_2_(PMe_3_)] with Ph_2_Mg(THF)_2_ eliminates benzene and forms **28**, while that with AlEt_3_ eliminates two equiv. of ethane and yields the dimeric species [Cp*IrPMe_3_(μ-AlEt)]_2_, **29**. Both **28** and **29** activate CO_2_, giving [Cp*Ir(PMe_3_)CO] as the TM-containing product.^62^

The TM···M distances in **27–29** are in range of the sum of the covalent radii, and the coordination at iridium in **27** is such that the Ir–Al bond is distorted away from the IrH_2_ plane by 37°.^62^ The geometries contrast with those of **30-W** and **30-Mo**. First reported by Wailes *et al.*^63^ and Storr *et al.*,^64^**30-W** has been characterised by X-ray diffraction and analysed by DFT methods.^65^,^66^ While the current understanding is that the two metals interact through a single hydride bridge to Al, there is limited data to support a non-negligible TM→M donation. Firstly, the HOMO on the TM fragment is calculated to be a metal-localised d-orbital-type suitable for electron donation. Secondly, the BX_3_ (X = F, Cl) adducts of the same TM fragments were calculated with the boron atom lying outside of the H–TM–H wedge.^66^

Although the gallium and boron analogues of **30** are yet to be isolated, ^1^H NMR data for a GaMe_3_/[Cp_2_WH_2_] admixture shows likely equilibrium between free compounds and a weakly bound adduct.^67^ These data are consistent with the known acceptor strength of the Lewis acids AlMe_3_ > GaMe_3_ > BMe_3_. Rhenium analogues of **30** were discovered by Wailes *et al.* using the metallocene [Cp_2_ReH].^63^ The potential for reversible H/D exchange between the hydrides and protons of the cyclopentadienyl ring of **30-W** has been highlighted and proposed to occur by a mechanism involving anchimeric assistance.^65^ Non-reversible intramolecular deprotonation of the cyclopentadienyl ligands is also well established, and often leads to high nuclearity species such as complex **31**.^68^–^71^

When [Cp_2_MoH_2_] was treated with ethylzinc bromide, **32** was isolated and presumed to derive from ZnBr_2_ formed from a Schlenk-type equilibrium.^72^ An η^2^:η^2^-bonding mode of the Mo hydrides is implied by the narrowing of the MoH_2_ wedge upon adduct formation, data that contrast those of **27**. A similar adduct, **33**, is formed from addition of a bis-aryloxy zinc solvate to a Rh trihydride complex.^73^

Reaction of a mixture of NbCl_5_, sodium cyclopentadienyl and zinc powder in THF under an atmosphere of CO, and subsequent treatment with NaBH_4_ gives **34**.^74^ The metal···metal distance in **34** is just within the sum of the covalent radii and there is a shift of the *ν*(CO) absorption upon coordination of the Lewis acid to [Cp_2_Nb(CO)H] (1960 to 1910 cm^–1^). Both findings mark a significant Nb → Zn interaction. In contrast, only a small shift in the *ν*(CO) absorption is seen upon reaction of [Cp_2_NbH(CO)] with AlEt_3_.^75^ Based on significant changes to the hydride resonance observed by ^1^H NMR data, the coordination of AlEt_3_ in [Cp_2_Nb(L)H·AlEt_3_] (L = CO, C_2_H_2_, PMe_3_) is proposed to occur through a μ-hydride ligand rather than a direct metal···metal interaction. Related reactions between a 1 : 1 mixture of [Cp_2_NbH_3_] or [Cp_2_TaH_3_] and [Cp_2_Zn] do not lead to adduct formation but cyclopentadiene elimination and formation of new species **35** and **36** both of which contain TM–Zn bonds.^76^,^77^ Tebbe and co-workers reported the d^1^ complex [(Cp_2_NbH_2_)_2_Zn] from the 1 : 2 reaction of Et_2_Zn with [Cp_2_NbH_3_].^75^ Higher nuclearity species **36–38** have been isolated from reactions of ruthenium polyhydride complexes or [Cp_2_MoH_2_] with main group alkyls (Fig. 9).^78^–^81^

**Fig. 10:**
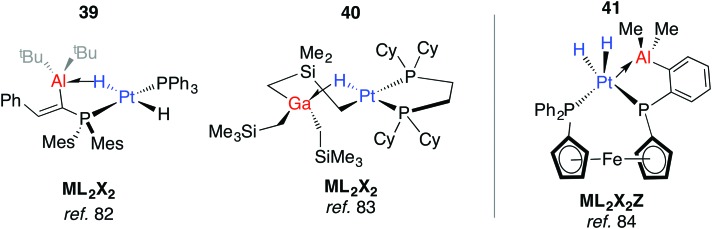
Adducts from H_2_ or C–H addition across a Pt→Al Bond.

In more recent work, Bourissou, Uhl and co-workers have shown that hydrogenation of an intramolecularly coordinated Pt→Al adduct leads to the heterobimetallic hydride **39** (Fig. 10).^82^ Calculations suggest that H_2_ addition occurs across the Pt→Al bond. There is precedent for this intramolecular coordination mode: Fischer and co-workers have reported the gallium adduct **40**.^83^ Related hydrogenation reactions of platinium diene complexes either bearing a Z-type Al ligand or in the presence of Ga^I^ co-ligands lead to the formation of heterobimetallic complexes bearing terminal hydride ligands on the transition metal (Fig. S1, ESI†).^46^,^84^ For example, **41** is formed upon hydrogenation of a Pt···norbornadiene precursor; it is currently unclear if the Pt→Al moiety plays a role in dihydrogen activation (Fig. 10).^84^

### Early transition metal adducts

**Fig. 11:**
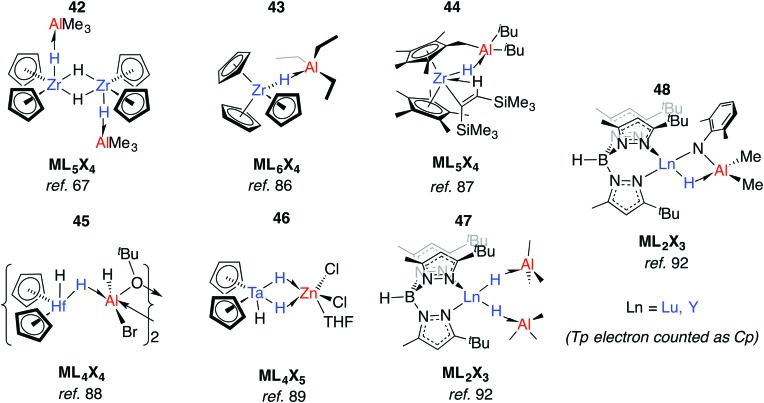
Early TM/main group adducts.

Relevant to Ziegler–Natta polymerisation, Zr–H→Al Lewis acid–base interactions have been known for some time.^85^ Coordinatively saturated, d^0^ heterobimetallic complexes may be formed by coordination of the parent hydride to a Lewis acid. For example, **42** was suggested by Wailes and co-workers based on ^1^H NMR experiments as early as 1972.^67^ Crystallographic characterisation of related adducts **43** and **44** containing inter- and intramolecular Zr–H–Al moieties was later reported.^86^,^87^ While there is less data for hafnium, the adduct **45** has been reported and is notable for the location of the Al centre outside of the H–Hf–H wedge. The position of the hydrides are unusual when compared to related group 4 metallocenes as is the 16-electron configuration of Hf.^88^ For group five analogues, [Cp_2_TaH_3_·MEt_*n*_] forms irreversibly for M = Al, Ga (*n* = 3) but reversibly for Zn (*n* = 2).^75^ Solid state infrared spectroscopy and X-ray diffraction data for a related complex, **46**, are consistent with formation of an adduct containing two Ta–H–Zn linkages forming an η^2^:η^2^-coordination mode, although NMR data suggest that only one bridging hydride is retained in solution (Fig. 11).^89^

Anwander *et al.* have investigated the reactions of yttrium and lutetium alkyl and amide complexes with aluminium hydrides.^90^–^93^ For example, lanthanide hydride bonds in **47** are capped and stabilised by Lewis acidic aluminium sites.^92^ This motif is not unique to **48**, and X-ray data has been collected on monomeric [Cp_2_Lu(μ-H)·AlH_3_·NEt_3_],^94^ and dimeric [Cp*Y(Me)(μ-H)AlMe_2_(μ-H)]_2_.^95^ Despite the coordinated Lewis acid, **47** displays reactivity consistent with a terminal hydride; deprotonation of 2,6-dimethylaniline gave complex **48**. Here, the imide linker supports an intramolecular Ln–H→Al interaction (Fig. 11).^92^

## 6. Multiply-bridged complexes (TM–H_*n*_–M, *n* > 1)

### Rare earth metal adducts

**Fig. 12:**
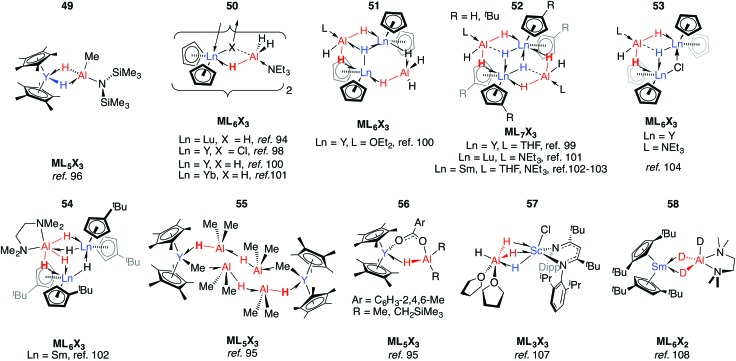
Coordination of aluminium hydrides to rare earth metal centres.

[(C_5_Me_5_)_2_YMe(THF)] reacts with HAl{N(SiMe_3_)_2_}_2_ allowing trapping of μ-H_2_-bridged **49**.^96^ The alane in this complex is strongly bound and addition of donor, including chelating, ligands could not effect separation of the heterobimetallic complex. The coordination mode in **49** is predated by those found in **50–55** reported by Bulychev and co-workers in the 1980s.^94^,^97^–^105^ In these latter complexes, the Al fragment and hydride source derive from either LiAlH_4_ or AlH_3_·L (L = NEt_3_, THF, OEt_2_), and either the chloride or hydride ligands take up μ^3^-coordination modes. Amongst the complexes [Cp_2_YH·AlH_3_L]_2_ (**50**), the Y···Al distances decrease, and Al–H stretching frequencies increase, across the series L = Et_2_O < THF < NEt_3_. The five-coordinate Al centres found in these complexes are notably different to the borate analogues, where the boron atom does not engage in coordination numbers higher than 4 and solvation occurs at the rare earth metal centre (Fig. 12).^106^

Alanes react with yttrocene hydride or carboxylate complexes to form Y←H–Al adducts (**55–56**).^95^ While investigating the salt metathesis of a β-diketiminato-supported Sc dichloride complex with LiAlH_4_, Piers and co-workers isolated the alane adduct, **57**.^107^ In **57**, both metals are six coordinate, and this is the only time a (μ-H)_3_ bridging motif seen in the absence of a late TM. Heterobimetallic hydrides of rare earth metals are not limited to those in which the heavy metal is in the 3+ oxidation. Bulychev *et al.* found that [(1,3-^*t*^Bu_2_-C_5_H_3_)_2_Sm] partially decomposes into an octanuclear aggregate of Sm^III^ and alane, when treated with AlH_3_ in the presence of TMEDA. Upon substitution of the alane for AlD_3_, however, **58** may be isolated: an apparent effect of isotopic substitution.^108^

### Group 4 metal adducts

**Fig. 13:**
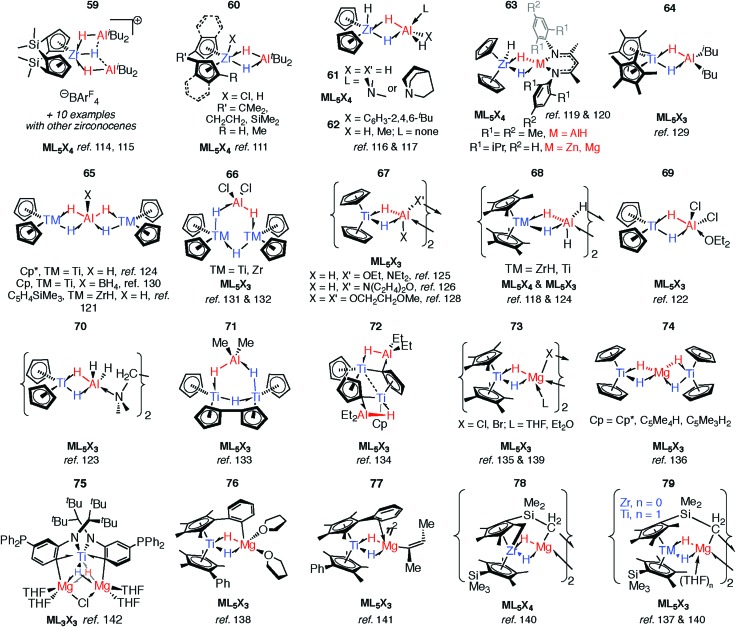
Group 4 complexes with multiple hydride bridges.

As part of understanding the role of methylaluminoxane in polymerization catalysis,^109^ Bercaw, Britzinger and others have reported NMR studies on hydride-bridged Zr/Al oligomers, trimers and dimers in solution (**59–60**).^85^,^110^–^112^ They concluded that binuclear structures of type **60** are only produced for *ansa*-metallocenes and inclusion of a terminal Me ligand on Zr causes decomposition. Cationic Zr- and Hf-analogues are known.^113^–^115^ It has been shown that {Zr(μ-H)_3_Al_2_}^+^ reacts reversibly with AlMe_3_ or ClAl^*i*^Bu_2_ to give dimethyl- or dichloro-bridged Zr/Al species (Fig. 13).^114^

An early example of a Zr–H–Al complex was published in 1997 by Raston and co-workers.^116^ Structurally and synthetically, **61** is logical extension of the work of Bulychev *et al.* (*vide supra*). These species are poorly soluble in all common organic solvents but THF, and require stabilisation by a base. Power and Wehmschulte added a super bulky aryl group (**62**) improving solubility and eradicating the need for base stabilisation.^117^ Base-free complexes have also been reported by Stephan and colleagues.^118^ NMR data and calculations for the monomers **62** suggest both fast intramolecular hydride exchange and an intermolecular exchange between the heterobimetallic complex and its homometallic parts in solution. Our group has also published an example of this type: soluble in toluene and hexane **63-Al** exists in equilibrium with the alane and [Cp_2_ZrH(μ-H)]_2_.^119^ In contrast, the homologues, **63-Mg** and **63-Zn** show no sign of dissociation into monometallic parts. This may be a result of tighter binding due to an increased ionic contribution to the donor–acceptor linkage (Fig. 13).^120^

Many of the Zr^IV^/Al heterobimetallic hydride complexes are synthetically accessible by salt-metathesis reactions of the parent zirconocene dihalide with aluminium hydride reagents.^121^ Similar reactions with titanium(iv) precursors commonly lead to isolation of products with Ti in the 3+ oxidation state.^122^–^128^ This is displayed most nakedly in the recent report of **64** by Beweries.^129^ Six-, five- and four- coordinate aluminium centres have been reported in {Ti_2_Al_2_H_8_}, {Ti_2_AlH_5_}, and {TiAlH_2_} cores respectively (**65–72**).^118^,^121^–^134^ There is only limited precedent for similar reduction chemistry occurring with Zr.^131^,^132^ The observed coordination modes of Al_*x*_H_*y*_ units in these and related group 4 complexes are common to heterobimetallics and are found in related Ta, Nb, Mn and Ru complexes (*vide infra*).

Substitution of the terminal hydride ligands on aluminium for halide, alkyl, alkoxide or amide has little influence on structure.^133^,^134^ Magnesium-based heterobimetallic complexes of group 4 metallocenes (**73–77**) can be prepared by reaction of the parent metallocene dichloride with either Grignard reagents or Mg^0^ powder in etheric solvents.^135^–^142^ For preparations in which a main group hydride is not used as a reagent the hydride ligands result from either: (i) C–H atom abstraction from the solvent (THF), (ii) β-hydride elimination group derived from a main group alkyl, or (iii) C–H bond activation of the cyclopentadienyl ligand (or its substituents). Ligand activation is common in these complexes and intramolecular deprotonation to form dianionic Cp ligands, including “tuck-in” complexes has been observed to lead to both diamagnetic TM^IV^ and paramagnetic Ti^III^ complexes (**72** & **78–79**).^126^,^133^,^134^,^143^,^144^

**Fig. 14:**
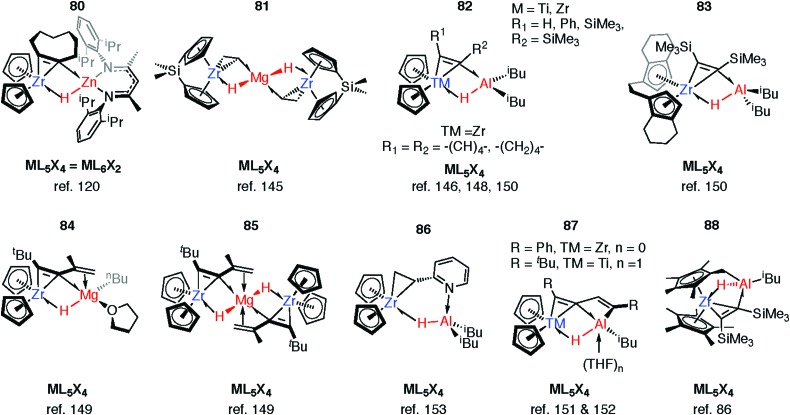
Coordination of M–H to Zr and Ti metallocyclopropene and metallocyclopropane complexes.

Reaction of **63-Zn** with an excess of 1,5-cyclooctadiene resulted in the on-metal transformation of the organic diene to an alkyne adduct, **80** (Fig. 14).^120^ This is an example of a heterobimetallic complex containing a planar, four-coordinate carbon and is related to the first confirmed zirconium-ethylene complex **81**, reported by Parkin and co-workers.^145^ Structurally related zirconium(iv) and titanium(iv) species are known in which the TM–H–M connection is supported by bridging metallacyclopropane or metallacyclopropene ligands (**80–88**, Fig. 14).^86^,^120^,^146^–^153^ A ketene analogue is also known which incorporates an oxycyclopropane ring.^154^ The structures of **84–87** are distorted to accommodate additional intramolecular binding interactions. Calculations have shown that electron donation from the bridging Zr–C bond to the Lewis acidic main group centre plays a key role in the stabilisation of these species (**82**) and explains the unusual geometry at the bridging carbon.^147^ A combination of computational methods (NBO and QTAIM) have led our group to describe the bridging ligand of **80** as a slipped metallocyclopropene.^120^

### Late transition metal adducts

**Fig. 15:**
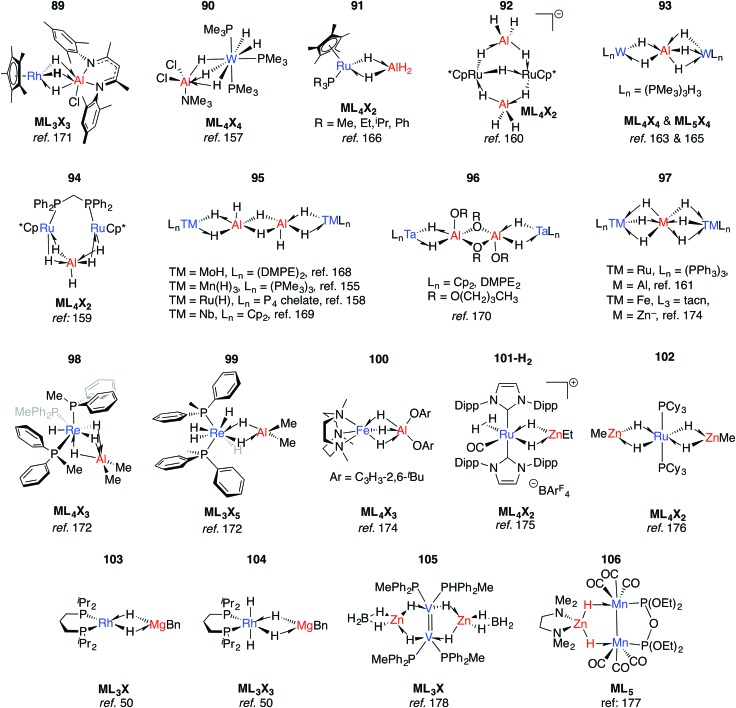
Late TM heterobimetallics containing multiple bridging hydrides (tacn = 1,4,7-triazacyclononane).

Multiply-bridged main group complexes (M = Mg, Zn, Al and Ga) of late transition metals have been known for more than 50 years. Much of the early work is limited by the accuracy of the spectroscopic methods of the period.^20^ Nevertheless, it has been clear for some time that the electronegativity difference between late TM and main group metals creates a significant ionic contribution to the bonding.^20^ Complexes **89–97** are mostly products of salt metathesis between lithium aluminohydrides and TM chlorides (Fig. 15).^155^–^171^

Common themes emerge in the coordination geometries of these complexes.^170^ Unlike the group 4 analogues described above, the (μ-H)_3_ bridge is a reoccurring feature. Geometries at aluminium are typically trigonal pyramidal (5-coordinate)^159^ or distorted-octahedral (6-coordinate). The high coordination number and the geometry of ligands around the TM centres provides coordinative saturation, and in most cases makes the existence of a metal–metal bond very unlikely. For **89**, this finding is in direct contrast to the data reported for the related Rh/Al species **17-Al** and **18** (Fig. 5).^171^

In solution many of these complexes are fluxional and provide time-averaged ^1^H NMR chemical shifts for bridging and terminal hydrides. In relation to this phenomenon, the complexes **98** and **99** reillustrate the ionic component of the bonding. The main group fragment of **98** and **99** is {AlMe_2_}^+^, which may exchange between different positions on the face of the {P_3_ReH_4_}^–^ and {P_2_ReH_6_}^–^ polyhedra.^172^ For comparison, [ReH_9_]^2–^ is well-known as the dipotassium salt.^173^

The low-spin ground state structure of **100** has been thoroughly investigated by computational methods.^174^ The frontier molecular orbitals of **100** do not support bonding between Fe and Al, and the authors present the complex as being dominated by a strong donation of electron density from a {Fe–H}^–^ fragment to {AlX_2_}^+^ (X = O-C_6_H_3_-2,6-^*t*^Bu). This conclusion is a good general insight into the electronic structure of the late-TM(H)_*n*_Al bonding, the dimeric examples of which are summarised in **95–96**.

An alternative synthetic approach to multiply-bridged main group complexes of the late transition metals is through the addition of H_2_ across an unsupported Ru–Zn bond. The Ru(H)_2_Zn moiety in **101-H_2_** discovered by Whittlesey and co-workers demonstrated good thermodynamic stability and survives extrusion of dihydrogen by heating *in vacuo* to form **101**.^175^ The hydride ligand *trans* to H_2_ in **101-H_2_** is protic and significantly closer to Ru, while the hydride *trans* to CO is hydridic and equidistant between the metals. NBO calculations show no sign of Ru–Zn interaction in either complex. However, a QTAIM calculation on **101** shows a Ru–Zn bond path and only one Zn–H bond. It can tentatively be concluded that the μ-H atoms in **101-H_2_** and **101** are intermediate and “flexible” between terminal and bridging character. Fischer and co-workers have reported the related complex **102**, which results from addition of 4.5 equivalents of ZnMe_2_ to a Ru/Al heterometallic complex prepared *in situ* by mixing [Ru(PCy_3_)_2_(η^2^-H_2_)(H)_2_] and [Cp*Al]_4_.^176^ Two decades before, Rh/Mg complexes **103** and **104** were prepared by Fryzuk, however their thermal instability precluded characterisation by anything other than ^1^H and ^31^P NMR spectroscopy (Fig. 15). A number of dinuclear (with respect to TM) complexes supported by chelating ligands or involving TM–TM bonding including **105–106** are also known.^177^,^178^

## 7. Discussion

An exhaustive account of the preparation and structures of heterobimetallic hydride complexes reported over the last half a century is presented above. During our own research in this area and through analysis of the material above, we have alighted upon recurring issues in the understanding this family of complexes. For the benefit of potential future studies, these issues are discussed below.

### (i) Structure and bonding

#### Sigma-complexes and oxidative addition

**Fig. 16:**
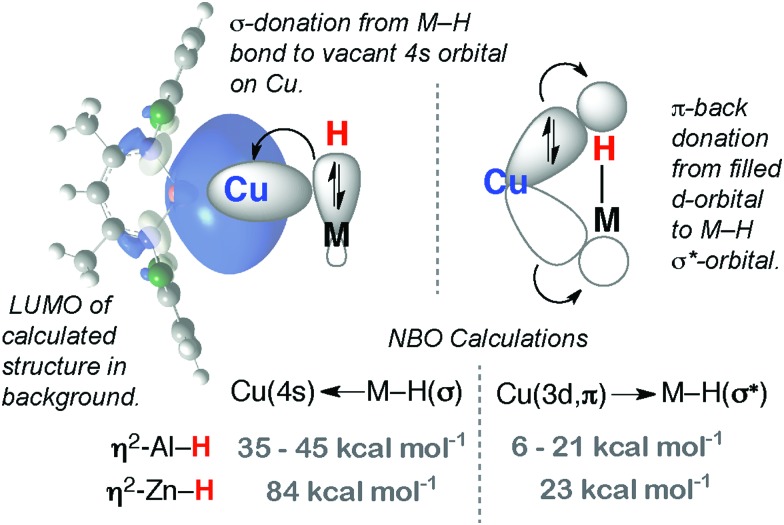
Bonding description of **1–3**.

The current understanding of the TM←H–M linkage (M = Mg, Zn, Al, Ga) in σ-complexes is that it is comprised primarily of a donor interaction from main group metal hydride to a vacant orbital of suitable symmetry on the transition metal fragment. There is limited data to support significant back-donation from the metal to the M–H σ*-orbital. For example, calculations on complexes **1–3** elucidate a donor–acceptor interaction with the 4s-orbital of the Cu d^10^ fragment acting as the acceptor: second-order perturbation calculations reveal only a small contribution from back-donation (Fig. 16).^27^

The model is supported by comparison of the carbonyl stretching frequencies of complexes of the form [(η^5^-C_5_H_4_Me)Mn(CO)_2_L] where L is an E–H or M–H bond. Listed in Table 1 these data show that σ-alane, σ-gallane and σ-zincane complexes are similar to those formed from four coordinate boranes such as H_3_B·NMe_3_. The data are consistent with limited back bonding into the M–H bond and contrast those of three coordinate σ-boranes and σ-silanes where increased back-donation to the E–H bond results in higher frequency carbonyl stretching frequencies.^37^ The domination of the σ-donation component of the bonding, and the importance of the ionic component to bonding, is readily understood by considering the difference in the Pauling electronegativity of the elements involved for coordination of E–H (Δ*χ*_p_: B = 0.18, Si = 0.32) and M–H bonds (Δ*χ*_p_: Mg = 0.92, Zn = 0.57, Al = 0.61, Ga = 0.41).

**Table 1:** Comparison of CO stretching frequencies in [(η^5^-C_5_H_4_Me)Mn(CO)_2_L]. BDI = (2,6-^i^Pr_2_C_6_H_3_CNMe)_2_CH. Adapted from ref. 37

L	*ν*_1_/cm^–1^	*ν*_2_/cm^–1^
H_2_	1982	1922
HBCat	1995	1937
HSiPh_3_	1983	1926
HGePh_3_	1965	1910
HSnPh_3_	1934	1925
H_3_B·NMe_3_	1918	1839
H–Al(H)BDI	1947	1879
H–Ga(H)BDI	1951	1886
H–ZnBDI	1937	1852

Considering the structures of the isolated complexes in Sections 2 and 3, it becomes clear that the well understood continuum between σ-complexes and oxidative addition products detailed for addition of E–H bonds to transition metals also applies to M–H bonds.

**Fig. 17:**
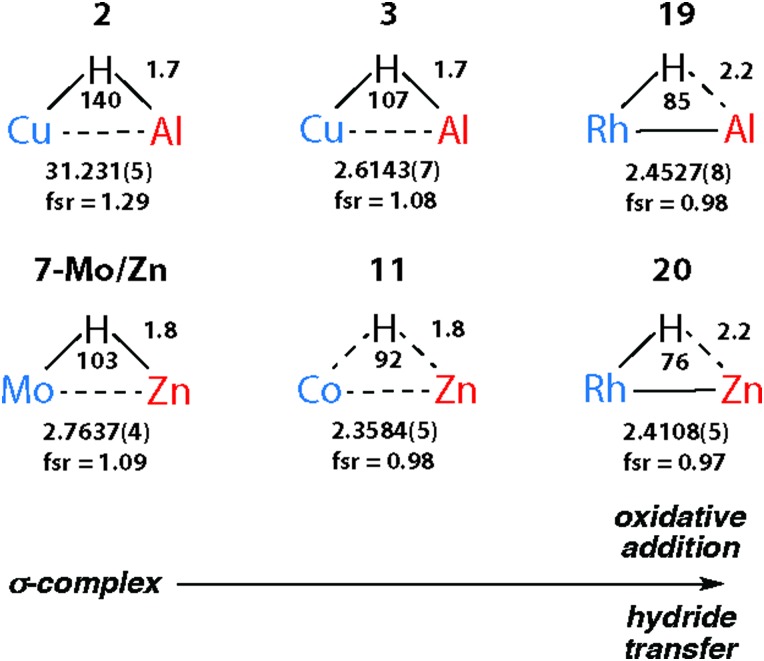
Comparison of key distances (Å) and angles (°) from X-ray (TM···M) structures of coordinated Al–H and Zn–H bonds. Trends confirmed by DFT studies.

The triangular TM–H–M unit is subject to structural changes as the electron density changes at the TM (Fig. 17). The formal shortness ratio (fsr) normalises the metal···metal distance and has been used to evaluate the intermetallic interaction in complexes containing two metals in close proximity. For data collected to date, this metric appears to conveniently describe the extremes of the reaction coordinate: σ-complexes (fsr approx. >1) and products of oxidative addition/hydride transfer (fsr approx. ≤1).^179^ One caveat of this approach is that short metal···metal distances can arise due to the geometric constraints imposed by multiple bridging ligands, to date the analysis has only been applied to molecules containing a single TM–H–M unit.

**Fig. 18:**
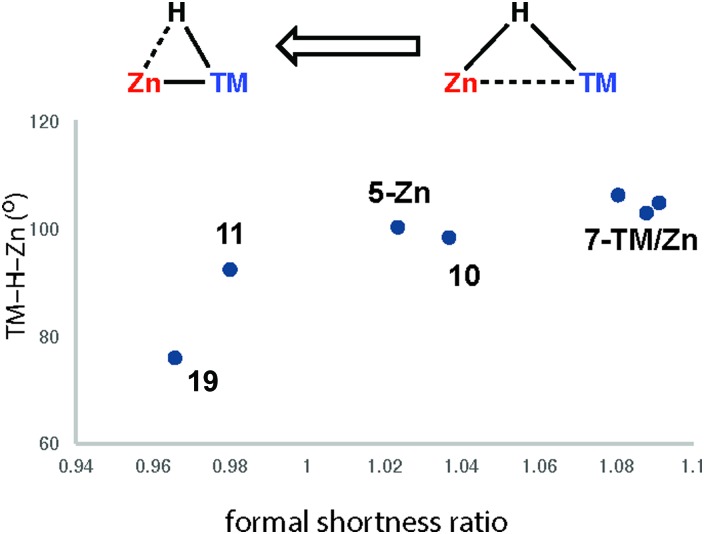
Reaction coordinate for the approach of a Zn–H bond to a transition metal. Adapted from ref. 37.

Very recently we have described the reaction coordinate for the addition of zinc-hydrides to transition metal centres.^37^ Through isolation of a series of different transition metal complexes and DFT studies we outlined a continuum which is characterised by the transfer of electron density from the breaking largely ionic Zn–H bond to forming, increasingly covalent, TM–H and TM–Zn bonds (Fig. 18). Only at very short TM···Zn separations does the Zn–H bond begin to lengthen significantly. The Zn–H bond varies between approx. 1.7–1.8 Å for σ-zincane complexes but increases dramatically to 2.2 Å for the product of oxidative addition. Similarly for σ-alane complexes the Al–H separation of 1.6–1.8 Å increases to >2.0 Å for products defined as oxidative addition. The breaking point of this bond appears to occur quite late along the reaction coordinate. While high quality and low temperature X-ray data is becoming increasingly well established for the location of hydride ligands in solid state structures, there is clearly significant error in the TM–H and M–H distances and associated TM–H–M angle. In the case of the analysis presented in Fig. 18, this concern is circumvented by performing DFT calculations to confirm the location of the hydride.

**Fig. 19:**
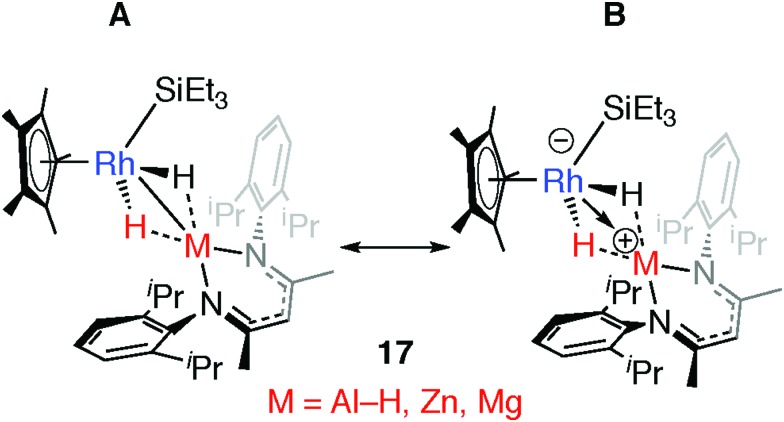
Extreme bonding description of **17** and related complexes. Adapted from ref. 43.

Comparison of a series of E–H and M–H complexes of a single transition metal fragment, {Cp*Rh(H)(SiEt_3_)} has allowed us to conclude that the ionic component to bonding remains important for the products of oxidative addition and is such that for M = Zn and Mg these complexes can accurately be described as a result of hydride transfer. The two extreme valence bond description of **17** and the related H–SiEt_3_ and H–Bpin complexes are represented in Fig. 19. DFT calculations show that the covalent contribution to the TM–M bond (Wiberg Bond Index) decreases along the series Mg ∼ Zn < Al < Si ∼ B while the charge on rhodium becomes significant for the Zn and Mg members of the series (NPA charge on Rh: Bpin = –0.03, SiEt_3_ =–0.06, Al(H)BDI = –0.11, ZnBDI = –1.01, MgBDI = –0.99).^43^ In combination the data suggest that the ionic contribution becomes more significant for the more electropositive elements.

Hydride transfer can also occur to generate complexes that do not possess a TM–M bond such as the tight ion-pair **20**.^45^ In the extreme case, charge separation could occur to form a completely ionic species, such as the salt [Zn(NH_3_)_4_][Cr_2_(μ-H)(CO)_10_]_2_.^180^

**Fig. 20:**
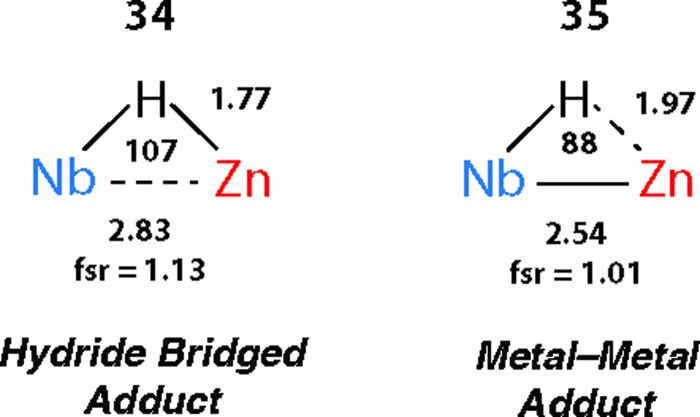
Comparison of the bond lengths (Å) and angles (°) in **34** and **35**. Figure adapted from ref. 76.

There are clear parallels between the zwitterionic valence bond description **B** and the neutral donor–acceptor complexes, TM→M–H. These species are connected parts of a continuum of bonding descriptions as are σ-complexes of the form TM←H–M and TM–H→M adducts. It appears that as the fsr provides some insight when comparing coordination complexes of the M–H bond to TM, it may also be of some use in considering coordination of the TM–H fragment to M. The bond distances and angles around the Nb–H–Zn moiety of **34** and **35** are compared in Fig. 20. The fsr ratio decreases and TM–H–M angle becomes increasingly acute when comparing a complex with a genuine TM–M bond (**34**) against that in which the primary interaction between the two metals occurs through a 3-centre 2-electron TM–H–M bond.

While, in general, the higher electronegativity of the main group element in silanes (and to an extent boranes) means that TM–H→ER_*n*_ bonding descriptions are less common, there is growing appreciation that these descriptions may be relevant in σ-silane and σ-borane chemistry. For example, an Ir–H^–^→SiR_3_^+^ interaction has recently been invoked to explain the electronic structure of an intermediate isolated during catalytic studies in the dehydrocoupling of silanes and alcohols.^181^ Related borane-capped transition metal hydrides, especially those generated by reversible addition across TM→B bonds are gaining increasing attention in catalytic hydrogenation processes.^182^

#### Multiply-bridged hydride complexes

**Fig. 21:**
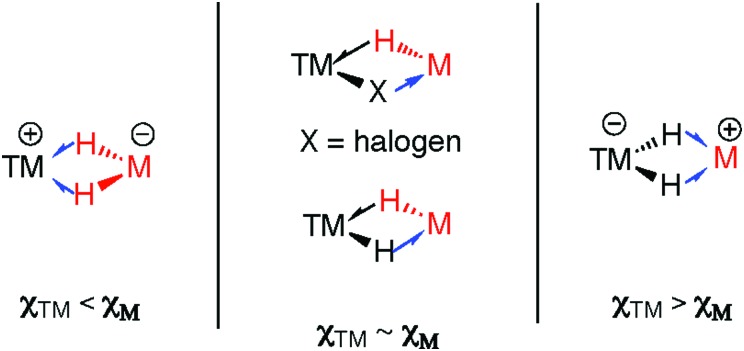
Limiting bonding descriptions in multiply bridged heterobimetallic complexes.

As the number of bridging hydride ligands increases so does the complexity of the structure and of the bonding descriptions. Wilkinson has commented: “*The similarity of…transition metal aluminohydrides to alanes suggest that, whereas it is correct to consider transition metal borohydrides as consisting of L*_*n*_*M*^+^*and BH*_*4*_^–^*moieties, similar models for aluminium analogues are not as accurate. It may be wiser to consider the complexes as being derived from donor–acceptor interactions*.”^20^ Although generally true for rare earth and group 4 complexes described in Section 6, many late transition metal adducts are described as ate-complexes formed of {TM–H_*n*_}^–^ and M^+^ fragments (Fig. 21).

**Fig. 22:**
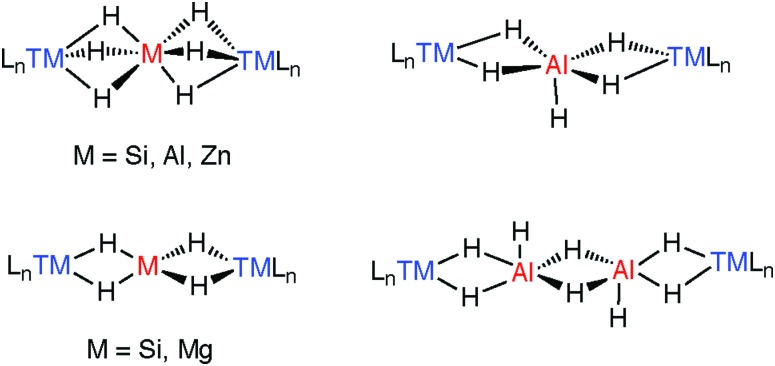
Coordination geometries of trapped MH_*x*_^*n*–^ units and comparison to silane chemistry.

A number of species have been isolated that can be considered coordination complexes of the trapped parent hydrides, these include MgH_4_^2–^, AlH_4_^–^, AlH_5_^2–^, AlH_6_^2–^, ZnH_6_^4–^, and Al_2_H_8_^2–^ the structural cores of which can be compared to similar complexes containing SiH_4_, SiH_6_^2–^ trapped between two transition metals.^183^,^184^ These species elucidate the general coordination modes found in multiply hydride bridged species (Fig. 22). More recently, we have found that the aggregation state of the complexes may be reduced by incorporating kinetically stabilising ligands on M.^27^,^37^,^43^,^119^,^120^,^171^

### (ii) Reactivity

The 3-centre 2-elecron TM–H–M moiety is surprisingly stable. The novel ground state structures found in heterobimetallic TM–H–M complexes are often assumed to herald unusual reactivity. This correlation is far from proven. Indeed, when it comes to reactivity, the understanding of the ground state structure is not enough. For example, a common feature of the data for the multiply-bridged hydrides is exchange between hydride positions in the solution phase.^175^

**Fig. 23:**
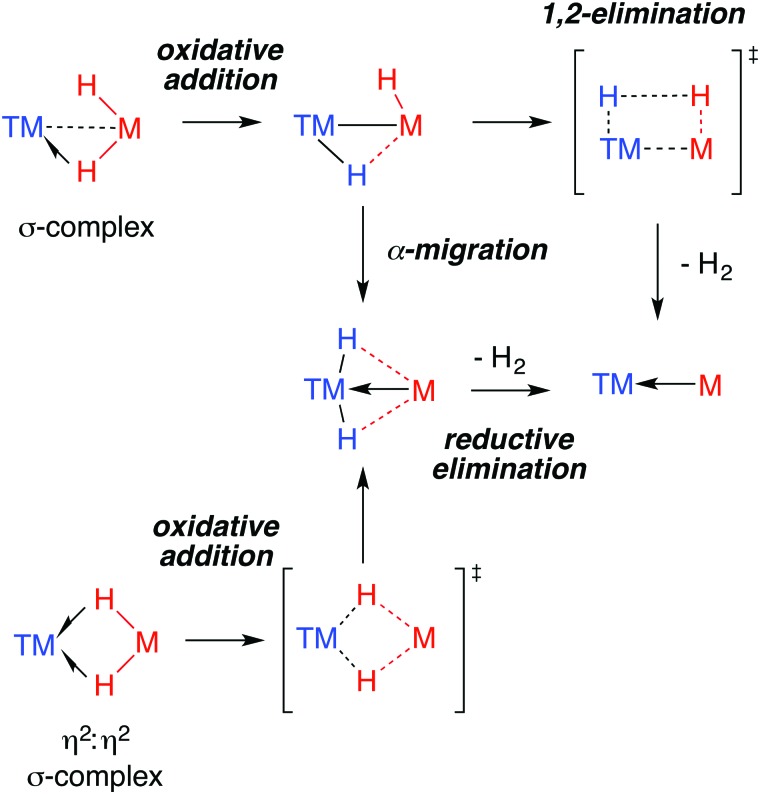
Proposed mechanisms for H_2_ elimination from heterobimetallic hydrides.

By far the most well understood reaction of TM–H–M groups are those which involve elimination of dihydrogen. Experimentally, while this reactivity has been observed for both Al and Ga complexes, examples of the latter dominate. Through the isolation of intermediates and kinetic analysis, a number of mechanisms for H_2_ elimination have been identified and are presented in Fig. 23. Both α-migration and 1,2-elimination of H_2_ across a TM–M bond have been used to rationalise the formation of Ga^I^ ligands in the coordination sphere of transition metals.

The microscopic reverse of the 1,2-elimination of dihydrogen from a TM(H)–M(H) unit is H_2_ addition across a dative TM←M bond. A handful of examples of this type of reactivity are known, albeit from adducts in which the roles of the metals in the Lewis acid–base adduct are reversed. For example, the addition of dihydrogen across Pt→Al and Ru→Zn bonds to form heterobimetallic complexes is known.^82^,^175^ It remains likely that similar reactivity will be discovered for TM←M adducts. This may well be foreshadowed by the existing observations of both inter- and intramolecular addition of carbon–hydrogen bonds across *in situ* generated TM←M (**16**) and TM–M (**20**, **31**, **44**) complexes.

By considering the properties of the TM–H–Zn complexes on the reaction trajectory presented in Fig. 18, DFT studies have shown that as the hydride is transferred from zinc it becomes less hydridic and more acidic.^37^ While the data would suggest that a rich acid/base chemistry may be possible with heterobimetallic hydride complexes, the reactivity of these species remains understudied. The same with insertion of unsaturated substrates (*e.g.* alkenes, alkynes, CO, CO_2_, carbonyls, *etc.*) into 3-centre 2-electron TM–H–M bonds: it remains unclear how this chemistry will compare to the well-studied reactions of transition metal hydrides.

### (iii) Potential in catalysis

Transition metal hydride complexes are known intermediates in the hydrogenation of unsaturated hydrocarbons, CO (Fischer–Tropsch), and CO_2_ along with numerous other small molecules.^185^–^187^ They also play a key role in hydrogenase enzymes and related catalysts for H^+^ reduction to H_2_ and have been invoked as (off-cycle) intermediates during a number of important polymerisation reactions including Ziegler–Natta catalysis.^85^,^109^

**Fig. 24:**
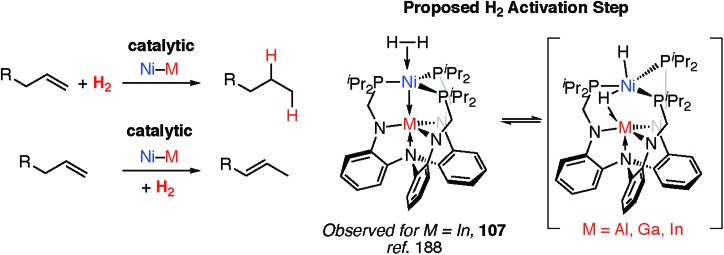
Catalytic alkene hydrogenation and isomerisation by heterobimetallic complexes.

To date however, defined catalytic reactions believed to involve heterobimetallic hydride complexes are limited. Systems that are catalytic in both the transition and main group metal are the most attractive. Lu and co-workers have reported the heterobimetallic complexes **107** as catalysts for alkene hydrogenation and isomerisation and demonstrated that the nature of the main group metal, M, effects the activity (Fig. 24).^188^ Dihydrogen activation by addition across a Ni→M bond has been proposed as a key step in hydrogenation catalysis.

The importance of transition metal hydrides to numerous aspects of catalysis is not up for debate. Whether or not heterobimetallic complexes will be able to offer advantages over existing monometallic systems remains an open question for the community. It is clear that substantial developments in the stoichiometric and catalytic reactivity of heterobimetallic hydrides need to be made. Nevertheless, the co-location of two metals by direct metal–metal bonds and/or bridging ligands offers two opportunities in catalysis: new fundamental reactivity, and fine tuning of selection events in known reactions.

The task for the current generation of chemists is to transcribe the known methods for preparation, and the structural understanding of, heterobimetallic hydride complexes into new reactivity. While progress in this area seems to have been made in stops and starts, given the renewed interest in heterobimetallics and main group complexes for catalysis, the challenge appears timely.^189^,^190^

## Supplementary Material

Supplementary information
