# Supplementary material for: Magnesium, zinc, aluminium and gallium hydride complexes of the transition metals
Source: Chem Commun (Camb). 2017 Jan 3;53(8):1348–65. doi: 10.1039/c6cc05702k (PMC5777540; doi:10.1039/c6cc05702k)
Supplement: Supplementary file 1 [file CC-053-C6CC05702K-s001.pdf]

Electronic Supplementary Material (ESI) for ChemComm.
This journal is © The Royal Society of Chemistry 2017

S1

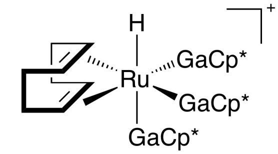

ref. 48

S2

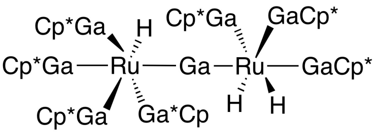

ref. 52

S3

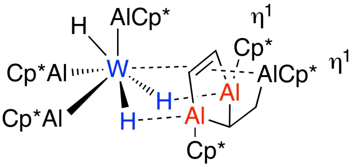

ref. 56

S4

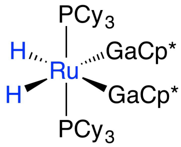

ref. 54

S5

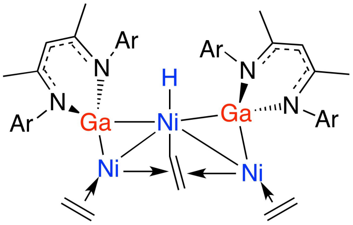

ref. 55

S6

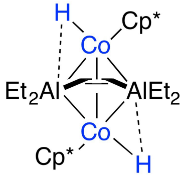

ref. 49

S7

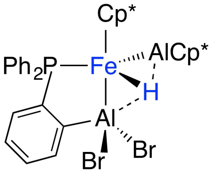

ref. 57

S8

$[Ru(ZnCp^*)_4(ZnMe)_4(H)_2]$

ref. 53

S9

$[(Cp^*AlCu)_6H_4]$

ref. 30

S10

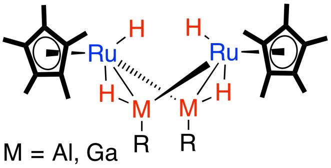

M = Al, Ga
R = η^1-Cp*

ref. 48

S11

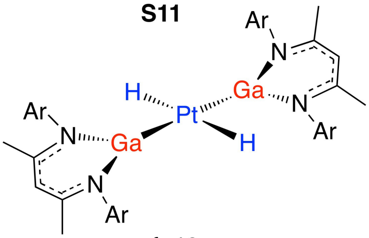

ref. 46

S12

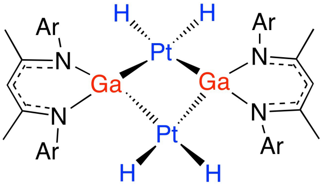

ref. 46

S13

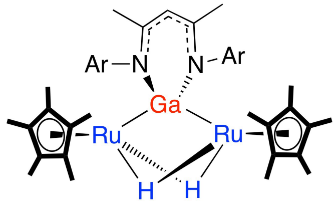

Ar = 2,6-$^iPr_2C_6H_3$
ref. 47

S14

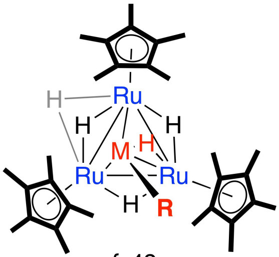

ref. 48

M = Al, Ga R = η^1-Cp*

S15

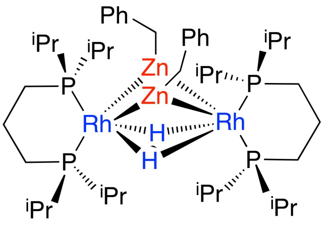

S16

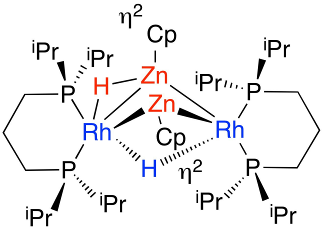

ref. 50 & 51
